# 2,4-Dichlorophenol Shows Estrogenic Endocrine Disruptor Activity by Altering Male Rat Sexual Behavior

**DOI:** 10.3390/toxics11100843

**Published:** 2023-10-07

**Authors:** Veronica Ferris Pasquini, Hector Hurtazo, Francisco Quintanilla, Martha Cruz-Soto

**Affiliations:** 1 Escuela de Ciencias de la Salud, Campus Querétaro, Universidad del Valle de México, Querétaro 76230, Mexico; v.ferrispasquini@gmail.com (V.F.P.);; 2 Programa de Gastronomía, Facultad de Filosofía, Campus Aeropuerto, Universidad Autónoma de Querétaro, Querétaro 76010, Mexico; franciscoqg_7@hotmail.com

**Keywords:** endocrine disruption, hormonal-induced sexual behavior, chlorophenols, estrogen activity

## Abstract

Chlorophenols (CPs) have been extensively used worldwide as a treatment to prevent the growth and proliferation of different microorganisms, mainly in the wood and farm industries. Chlorine has been used for water disinfection, and phenol groups are water contaminants; these two groups can react with each other to form species such as 2,4-dichlorophenol (2,4-DCP). 2,4-DCP is still used as an herbicide in many countries such as Mexico. CPs have been largely analyzed, like bisphenol A, for their probable endocrine-disrupting effects in humans and aquatic animals. We still do not understand whether these endocrine responses can be manifested as an impairment in sexual behavior in rodents. With the present toxicology study, the endocrine-disrupting effects of 2,4-DCP on male sexual behavior were investigated. Sexually naïve male Wistar rats were used to assess the endocrine-disrupting effects of 2,4-DCP. The rats were divided into two groups: one control group and one experimental group that was administered 1.25 mg/day of 2,4-DCP for 45 days. After completing treatment, the male sexual behavior of the rats was evaluated. The results of this investigation demonstrated that 2,4-DCP affected male sexual behavior. A decrease in mount latency, intromission latency, and post ejaculation period compared with the control animals was found.

## 1. Introduction

The endocrine system is responsible, to a certain degree, for an organism’s homeostasis. It achieves this by releasing hormones in low concentrations at very specific time frames during development and adulthood. Exogenous substances, which mimic hormonal activity and interfere with hormone signaling, are described as endocrine disruptors [1,2,3]. Endocrine-disrupting chemicals may interact with androgen and estrogen nuclear receptors and thereby alter hormonally induced behaviors [3,4]. Evidence exists demonstrating that estrogen may promote the development of estrogen-dependent tumors after the tumor has been initiated by other factors. Such factors include endocrine-disrupting chemicals with estrogen-like activities, otherwise known as xenoestrogens. These chemicals, along with their epigenetic molecular mechanisms, can participate in the multifactorial process of carcinogenesis. Scholars think that the high incidence of breast cancer worldwide can be attributed to the involvement of environmental pollutants such as xenoestrogens, in addition to the already well-known risk factors for this disease [5,6].

The various pesticides, rooting agents, formation of somatic embryos in soybean, and herbicides currently used are predominantly endocrine-disrupting chemicals [7]. Further, phenols and chlorines may react to form 2,4-DCP during drinking-water disinfection [8]. Chlorophenols are thereupon present in both the food chain and aquatic environment. As such, they are a strong concern for human health, wildlife, and for many ecosystems.

Endocrine disruption has been shown to negatively affect humans, wildlife, and domestic animals. The adverse effects caused by low dose exposure to these chemicals have highly impacted many processes including steroid hormone synthesis and secretion, reproduction, development, sexual behavior, metabolic stability, and glucose metabolism; some of these processes have displayed epigenetic inheritance patterns of the detrimental effects resulting from endocrine disruption [9,10,11,12]. The administration of 2,4-DCP affects the endocrine system, as has been determined by using different toxicological animal models [8]. There is evidence of the estrogenic effects of 2,4-DCP in zebrafish, demonstrating alterations in steroid hormone synthesis pathways and gonadal differentiation causing female differentiation of larvae [13]; additionally, 2,4-DCP has been shown to impair mouse oocyte sperm binding to the zona pellucida [14].

The effects of endocrine-disrupting chemicals are tissue-specific. Brain tissue has also been shown to be affected, as demonstrated by the results of previous works from Steinach, who exhibited the role of estrogen in male sexual behavior [15], and from Walker, who reviewed how steroids regulate sexual behavior [3]. The lipophilic nature of some endocrine-disrupting chemicals, as determined by the results of in vivo studies, implies that they can cross membranes, including the blood–brain barrier, and can possibly reach the sexual behavior control areas of the brain, such as the medial preoptic area and olfactory bulb [3,12,16].

Low dose human exposure to endocrine-disrupting chemicals during different developmental stages, from being a single cell to adolescence and adulthood, affects brain cells and circuits that depend on estrogen; this can result in neurological health problems including anxiety, learning and memory difficulties, and behavior alterations, as well as sexual selection [17,18]. The results of experimental toxicological studies on rodents have shown the importance of studying sexual behavior to understand sexual dysfunction [19,20]. These studies also serve to analyze the effects of endocrine-disrupting chemical exposure on mating behavior [21]. Ovarian hormones such as estrogen and progesterone have been shown to be implicated in the sexual behavior of female rats [22]. Copulatory behavior has also been shown to be regulated by estrogen in male rats [23].

Currently, it is well accepted that humans and wildlife are frequently exposed to endocrine-disrupting chemicals in lower doses than those used in animal studies. Nonetheless, studies in humans, particularly in females, have shown many different effects of these xenobiotics; receptor expression can be altered in brain areas that are important for sexual behavior and reproduction, notably with perinatal and sex steroid receptors; epigenetic mechanisms of action have also been seen [3,24,25,26,27].

In 2009, the Endocrine Society released the first scientific statement about the possible health threat that endocrine-disrupting chemicals may have and how these chemicals alter endocrine homeostasis, primarily studied in toxicological animal models [28]. The highlights of the first report included how endocrine-disrupting chemicals affect male and female reproduction in humans and in different animal models. One of the main recommendations of this report was to increase both basic and clinical research to raise awareness about the consequences of exposure to these chemicals. In 2015, the Endocrine Society released the second scientific statement; this report confirmed the importance of investigating the effects of these chemicals on health and disease, analyzing the low-dose effects of these substances in humans, and creating strategies on how to decrease their use and exposure. Additionally, it encouraged the scientific community to identify new chemicals with possible endocrine-disrupting activity [29]. The aim of the present work was to characterize in a toxicological rat model an endocrine-disrupting chemical with estrogenic activity, manifested by an alteration in hormonal male mating behaviors, such as mounting, intromission, post ejaculation time; effects on olfactory preference and partner preference were also investigated.

## 2. Materials and Methods

### 2.1. Animals

Animal care was provided by the Universidad del Valle de México (UVM) vivarium staff, complying with Official Mexican Standards and Regulations for the Technical Specifications of Production, Care and Use of Laboratory Animals [30]. Animal care was also approved by both the University Research and Bioethics Committees. The animals were housed 5 per cage at the UVM vivarium, which had two floors, each with a separate light/dark cycle. Females were housed on the first floor and males were housed on the second floor. Water and food (5001 Rodent Diet, Lab Diet DIMEBA Mexico, MI, USA) were available ad libitum.

#### 2.1.1. Males

Sexually naïve male Wistar rats weighing between 300 g and 350 g from a local colony were kept under a reversed 12 h light/dark cycle (lights off at 0700 h and on at 1900 h). They had free access to food and water. The animals were divided into experimental and control groups. Beginning at age 13 weeks, when late adolescence is set, the experimental group received 1.25 mg at a volume of 200 uL of 2,4-DCP (Sigma-Aldrich, Burlington, MA, USA, NAIS Mexico) daily for 45 days. The control group did not receive any substance administration.

#### 2.1.2. Females

Sexually naïve female Wistar rats weighing between 250 g and 300 g from a local colony were kept under a regular 12 h light/dark cycle (lights on at 0700 h and off at 1900 h). They had free access to food and water. The animals were divided into experimental and control groups. Each group consisted of two further subgroups: ovariectomized and intact. Those comprising the ovariectomized subgroups received ovariectomy surgery before 9 weeks of age, before the peripubertal stage. Those comprising intact subgroups did not receive any surgical procedure or manipulation. Beginning at age 13 weeks, the experimental groups received 1.25 mg at a volume of 200 μL of 2,4-DCP (Sigma-Aldrich, NAIS Mexico) daily for 45 days. The control groups did not receive any substance administration. All experiments were carried out in accordance with General Health Law Regulations regarding Health Research established by the Mexican Health Ministry. This includes limiting the number of animals used for the experiment and taking measures to minimize their suffering.

### 2.2. Surgery

Ovariectomies were performed under ketamine anesthesia by intraperitoneal injection at a dose of 4.5 mg/kg. A ventral approach was employed [31], using small sterile scissors to make a mid-ventral incision. The abdomen was entered via the linea alba and the uterine horns, ovaries, and surrounding fat were exteriorized by gentile retraction. The ovaries were isolated by ligation of the distal uterine horns with catgut and removed before returning the horns to the peritoneal cavity. The muscle and skin layers were closed separately with sterile sutures, each in a simple continuous pattern. A high degree of aseptic technique was maintained throughout the procedure. Postsurgical recuperation was monitored daily.

### 2.3. Administration of 2,4-Dichlorophenol

Experimental rats received enteral administration of 2,4-DCP at a dose of 1.25 mg/day via gavage using corn oil as the vehicle. Using the under-the-shoulder-grip restraint technique [32], administration was performed with a 24 F pediatric nasogastric tube once daily for 45 days, starting at 13 weeks of age. The volume administered was 200 μL. The syringe and gavage tube were filled to the correct amount, and the external coating was removed to ensure correct dosing. The animal was then restrained, and the gavage tube inserted into the left side of the mouth along the hard palate until reaching the back of the mouth. With gentle pressure of the tube, the head of the animal was tilted back towards the spine to allow esophageal alignment with the stomach. The respiration of the animal was then observed to ensure normal breathing and the absence of erroneous placement in the trachea. If respiratory distress was identified, the tube was removed, and the procedure was repeated.

After administration, the feeding tube was gently removed, and the animal was returned to its cage. Animals were monitored for 10 min post-administration to observe for potential complications.

### 2.4. Male Sexual Behavior

Stimulus females were ovariectomized (ovx) to be able to manipulate the phases of their estrous cycles for the behavior tests. Induction of estrus (heat) was achieved by hormone treatment. Each rat was injected with 25 μg of estradiol benzoate (EB) (Sigma, St. Louis, MO, USA) 48 h prior to mating tests, as well as 1 mg of progesterone (Aldrich, St. Louis, MO, USA), 4–6 h prior to mating tests.

After 45 days of daily administration of 2,4-DCP to male rats, male sexual behavior was evaluated as described by Hurtazo and Paredes [33]. Male sexual behavior tests were recorded in a wooden cage that measured 40 cm × 60 cm × 40 cm with the front wall made of glass to allow for observation. The stimulus female was first placed in the cage, and after 5 min, the male subject was introduced and observed. The following parameters were recorded: (1) mount and intromission latencies; (2) the number of mounts and intromissions; (3) ejaculation latency. Animals were observed until the end of the postejaculatory interval or, if the subject did not ejaculate, for 30 min after being placed in the observation cage. A total of three tests were performed for each male subject at weekly intervals.

### 2.5. Olfactory Preference

One week after the sexual behavior tests, olfactory preference was evaluated as described by Hurtazo and Paredes [33]. The estrus bedding was collected from groups of six ovx females treated with estradiol benzoate 48 h prior to the olfactory preference tests, as well as progesterone 6 h prior to the tests. The anestrous bedding was collected from groups of six ovx females that had been injected with oil 48 h prior to the olfactory preference tests, as well as 6 h prior to the tests. Soiled bedding was used in the tests within 4 h of collection. Fresh sawdust was used as control bedding.

Each subject was tested for a duration of 10 min on two separate days for their preference to approach and investigate different olfactory stimuli. Subjects were placed individually in a wooden cage that measured 40 cm × 60 cm × 40 cm, and enclosed were three bowls. Each bowl contained either clean, estrus, or anestrous bedding and was placed in different corners of the observation cage. At the time of the test, the observer was unaware of which bowl corresponded to which type of shavings. The following criteria were used to define the subject’s investigation of each bowl: the head of the subject had to be over the bowl, introducing its nose into the bedding at an angle of 45° from the bowl’s horizontal plane. The time spent in each bowl was recorded using four timers: three recorded the time spent in each bowl, and the fourth recorded the 10-min test duration. The data from the two tests were combined.

### 2.6. Partner Preference

One week after the olfactory preference tests, partner preference was evaluated as described by Romero-Carbente [34]. Tests were recorded in a wooden cage. The cage contained 3 compartments of the same color and texture, and the middle compartment communicated with the lateral compartments through a sliding door. The lateral compartments measured 10 cm × 10 cm on each side. A stimulus animal was placed in each lateral compartment and was wearing a harness that attached to the rear of the cage with a flexible rope, limiting their action radius within their compartment. In this context, a stimulus animal refers to a potential mate or partner to assess the preference and social behavior of the test subject. A sexually active male was placed in the harness of one lateral compartment, and a receptive female was placed in the harness of the opposite lateral compartment. Stimulus animals were still able to display coital behavior with the harness secured.

Each subject was tested for a duration of 10 min on one occasion for their preference to interact with the stimulus animals. The preference score was calculated by dividing the amount of time spent with the receptive female by the sum of time spent with both the receptive female and sexually active male. A lack of preference is indicated by a score of 0.5. Preference for the receptive female is indicated by a score of >0.5. Preference for the sexually active male is indicated by a score of <0.5.

### 2.7. Vaginal Smears

#### 2.7.1. Collection and Processing

Vaginal cytology samples were collected over a period of 7 weeks. The samples were obtained daily at 7:00 am, shortly after the lights were turned on after each 12-h dark cycle. Collection was performed by vaginal lavage [35]. One-piece plastic Pasteur pipettes with 2 mL capacity were used. A different pipette was used for each rat, and one sample was collected per animal. To perform the vaginal lavage, 0.2–0.5 mL of saline was drawn into the pipette, and then inserted into the vaginal orifice of the rat to approximately 5–10 mm of depth. The saline was flushed into the vagina and back out 2 times, and then a drop of the sample was placed on a slide and was allowed to air dry. One drop of methylene blue was then applied to each sample, which was then covered with a slide cover glass slip. This process was repeated for each rat.

#### 2.7.2. Slide Evaluation

A light microscope with Köhler illumination was used to identify cell estrous stages with 10× and 40× objectives for each sample [35]. Slide examination was performed by two evaluators, utilizing practice slide sets for comparison. The evaluators were aware of the preceding day’s smear stages. Estrous stages were recorded daily in a calendar chart.

### 2.8. Blood Sample Collection and Hormone Assays

In order to identify systemic changes in estradiol and testosterone concentrations that could elicit an effect on behavioral responses, blood samples were taken. Procedures were carried out under ketamine anesthesia by intraperitoneal injection at a dose of 4.5 mg/kg. Peripheral blood was collected by heart puncture using an insulin syringe. Animals were sacrificed thereafter following blood sampling.

After centrifugation, blood serum was kept frozen for further estradiol and testosterone assay by chemiluminescence.

## 3. Statistical Analysis

### 3.1. Male Sexual Behavior

Male mating behaviors were analyzed using a Student´s *t*-test to compare the means of treatment and control groups for the following parameters: mount latency, intromission latency and post ejaculation period. For the mount latency, normal distribution was observed; for intromission latency, the treatment group data were not normally distributed and the Mann-Whitney U test was used; and for the post ejaculation period, normal distribution was also observed.

### 3.2. Olfactory Preference

Levene’s test was used to assess the homogeneity of variance among the three differ-ent olfactory stimuli from the bedding explored. A Kruskall-Wallis test was then used to assess the difference between the olfactory stimuli. Finally, a Bonferroni post hoc test was used to determine where the differences between the group means were situated.

### 3.3. Partner Preference

A paired t-test was conducted to compare the duration of time spent with each stimulus animal for the course of the test. Normality was verified with a Shapiro-Wilk test.

## 4. Results

### 4.1. Male Sexual Behavior

The execution of copulatory behavior is a learned conduct. The rat progresses from naïve to expert as copulation experience increases. Control animals showed the expected sexual behavior when exposed to receptive females. The treated animals showed a reduction in time to execute the copulation behaviors compared to their control counterparts, and this time decreased with each subsequent copulation experience.

**Figure 1:**
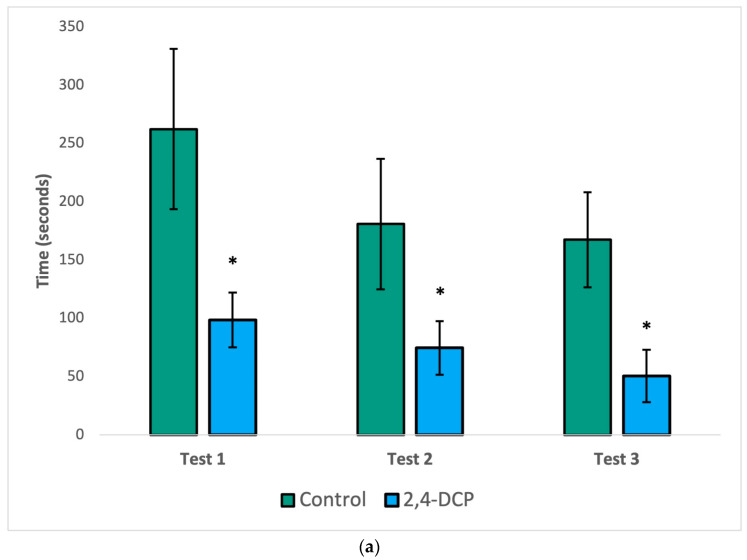

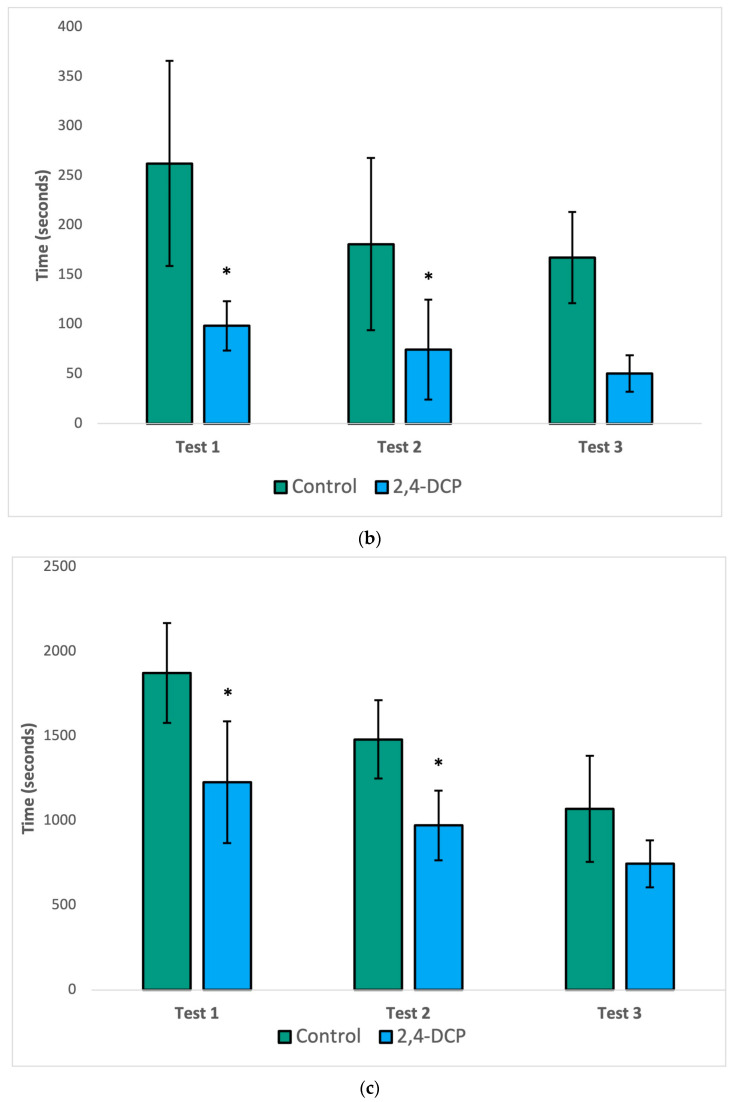
Sexual behavior test. Decreased mount latency (**a**), intromission latency (**b**), and post ejac-ulatory period (**c**). *n* = 8, (*) *p* < 0.05, error bars: +/−1 SD.

As seen in Figure 1, the mount latency (a), intromission latency (b), and post ejaculatory period (c) of 2,4-DCP-treated males were significantly reduced compared to control animals. In all three figures, a general decline in the parameter being analyzed was observed in both groups with each subsequent test. This is to be expected because as each test is repeated, the sexually naïve male rats gain experience and become more efficient in mating behaviors. Despite this general decline, statistically significant differences were still appreciated in all three tests for mount latency, as well as in the first two tests for intromission latency and the post ejaculatory period.

### 4.2. Olfactory Preference

In the Olfactory Preference test, we initially assessed the homogeneity of variance among the three types of olfactory stimuli using Levene’s test. Subsequently, we conducted a Kruskall-Wallis test, which revealed significant differences among the group means, indicating that there are statistically meaningful distinctions between the three groups.

**Figure 2:**
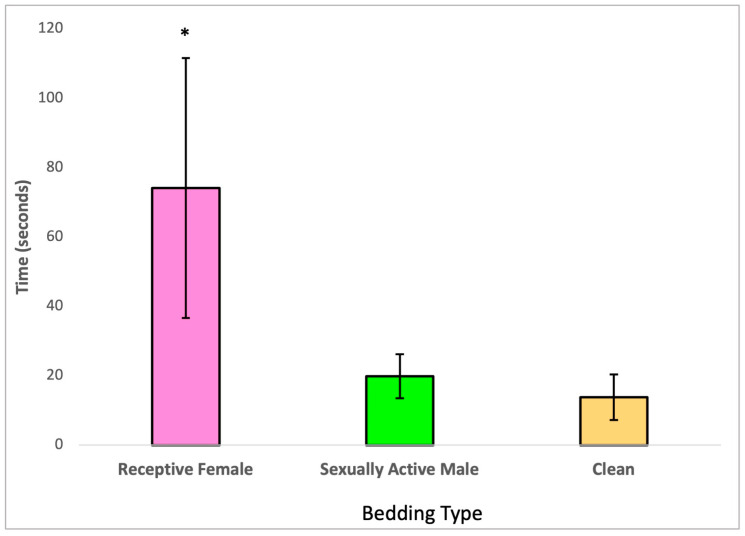
Olfactory preference of 2,4-DCP-treated males, *n* = 8, (*) *p* < 0.05, error bars: +/−1 SD.

Upon further analysis with a Bonferroni post hoc test, it became evident that the males treated with 2,4-DCP exhibited a preference for estrus bedding (soiled bedding from receptive females) over both anestrous bedding and soiled bedding from sexually active males, as illustrated in Figure 2. Additionally, this analysis indicated that the time these treated males spent sniffing soiled bedding from sexually active males compared to clean bedding was not significantly different.

### 4.3. Partner Preference

**Figure 3:**
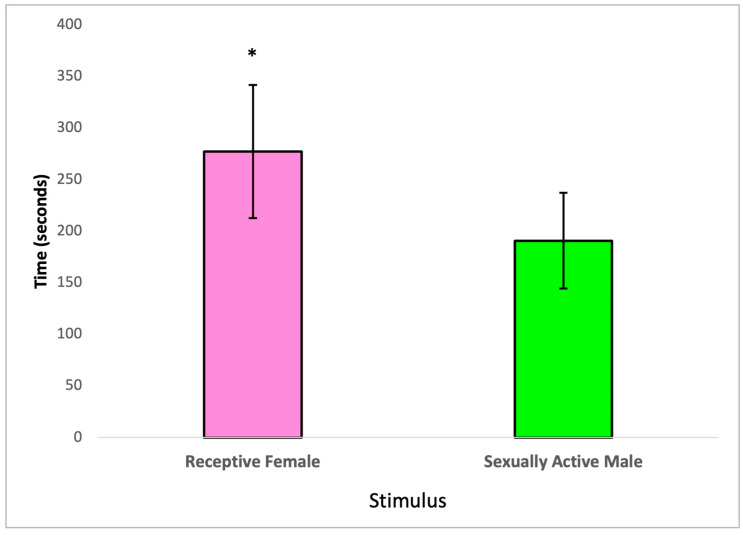
Partner preference of 2,4-DCP-treated males, *n* = 8 (*) *p* < 0.05, error bars: +/−1 SD.

In this section, we examined the partner preference behavior of treated male subjects, taking into account the social nature of rats. Specifically, we assessed whether male rats exposed to 2,4-DCP would spend more time with receptive females or sexually active males. Our analysis, conducted using a paired t-test, revealed statistically significant differences in the time 2,4-DCP-treated males spent with receptive females compared to the time they spent with sexually active males. These findings are visually represented in Figure 3.

### 4.4. Blood Sample Collection and Hormone Assays

Plasma testosterone and estradiol concentrations from male and female controls as well as 2,4-DCP-treated animals were measured in order to identify an influence on behavioral responses. It was confirmed that there were no changes in systemic concentration of these two steroids. Therefore, no interference with the results was assumed (Table 1).

## 5. Discussion

Studies in rats have demonstrated that normal sexual behavior is initiated by an increase in testosterone, which then activates aromatase in the brain, and as a consequence of this activation, estrogen is synthesized [36]. It has also been demonstrated that a reduction in local estrogen concentrations in the brain leads to an inhibition of sexual behavior. Therefore, it can be assumed that an endocrine disruptor with estrogenic effects, and the ability to cross the blood brain barrier, will increase local estrogen concentrations in key areas of mating behavior in the brain. The repercussions of this increase in local estrogen will be apparent in changes to typical sexual behavior.

Overall, our findings demonstrate that 2,4-DCP is able to infiltrate brain areas responsible for controlling male sexual behavior. In order to identify systemic changes in estradiol and testosterone concentrations, which could have an influence on behavioral responses, peripheral blood tests for estrogen and testosterone were performed; these confirmed that there were no changes in the systemic concentration of these two steroids and thus no interference with the results was assumed (Table 1). The reported oral LD50 of 2,4-DCP is 2830 mg/kg in rats. In the present toxicological study, we used an oral dose not near the LD50. Although human EDC exposure doses are usually low, the present animal toxicological study will provide useful information for EDC risk assessments in humans.

Sexual reproduction in rats involves certain sexually differentiated behaviors in both females and males. Male behaviors are initiated by olfactory cues from the presence of a receptive female, followed by the sexual motivation of the male to engage in sexual conduct, ultimately leading to copulation [21].

Endocrine disruptor-treated animals were compared to their control counterparts with three hormonal sexual behavior tests. In the first test of male sexual behavior, when a receptive female is present, the parameters mount latency, intromission latency, and post ejaculatory period were significantly reduced compared to control animals, as shown in Figure 1. These findings indicate that control animals show the expected sexual behavior when exposed to recipient females and that treated animals show more efficient sexual behavior; the males behave as experts, due to increased local estrogen concentrations in the brain.

Odors are of great importance in partner preference in both male and female rats [16,37]. Our findings from the second sexual behavior analysis, the olfactory preference test, demonstrated that 2,4-DCP-treated males maintained their preference for olfactory cues from receptive females, as opposed to cues from sexually active males and clean cage bedding, shown in Figure 2. One limitation of this study was that this test was not performed against non-sexually active control males; however, it would have been interesting to see if there was any difference in the degree of interest of female olfactory cues between treated and control groups.

The third sexual behavior analysis, the partner preference test, which analyzed advancement and consummation of sexual activity [16], demonstrated that 2,4-DCP-treated males maintained their preference for receptive females rather than for sexually active males, as shown in Figure 3. This is demonstrated by a significantly greater time spent with receptive females over sexually active males. Both olfactory and partner preference tests show that treatment with the endocrine disruptor 2,4-DCP in male rats may increase local estrogen concentration in the brain to elicit and maintain male sexual behavior without altering copulation motivation or partner preference. In prior studies, it has been shown that gonadal hormones participate in sexual preference and that testosterone aromatization is essential in male rats to elicit the masculinization of sexual behavior [38]. An advantageous aspect of this study is that it demonstrated how 2,4-DCP provided the estrogen needed to favor the execution of masculine sexual behavior in male rats, with no apparent aromatization of testosterone required.

Another strength of this study is that treated males were more efficient in executing the sexual behavior than their control counterparts, as they may have experienced a decreased sexual satiety shown by a diminution of the post ejaculation period [39] as a consequence of the increased local estrogen concentration and a possible activation of endocannabinoid and dopaminergic-key brain areas which regulate sexual behavior motivation.

Further analyses are suggested to elucidate epigenetic changes from exposure to 2,4-DPC and any molecular changes in areas of the brain that control sexual behavior after treatment with 2,4-DCP. This could possibly involve central changes in the expression of aromatase as well as that of steroid hormone receptors. Additionally, research suggests that sexual behavior causes plastic changes in the brain, and repeated sexual activity in males can be related to opioid plasticity [40]; our findings of treated males executing sexual behavior in a more experienced way than controls may implicate the development of plastic changes in their brains related to endocrine disruptor exposure.

## 6. Conclusions

The effects of an endocrine disruptor with estrogenic activity were evaluated by means of three sexual behavior tests. In the first, copulatory activity was assessed by mount latency, intromission latency, and post ejaculation time. Our results have confirmed that 2,4-DCP is effectively an endocrine disruptor with estrogenic activity; male rats treated with this chemical were more efficient in performing sexual behaviors than their control counterparts. The other two sexual behavior tests showed that (1) 2,4-DCP treatment has no effect on partner preference: treatment did not have an influence on males choosing receptive females, and (2) that a clear distinction of olfactory clues were perceived by treated males, showing they were motivated enough to perform and consummate hormonal sexual activity. Modulation of sexual behavior indicates that this molecule is lipophilic enough to cross membranes and to accumulate in local brain areas that control sexual behavior with an apparent lack of modification in systemic hormone concentrations.

Further studies are required to determine the neural and molecular mechanisms involved in the sexual behavior effects demonstrated in response to 2,4-DCP administration, particularly to illustrate possible changes in aromatase expression and steroid hormone receptor expression at the molecular level in the key brain areas controlling sexual behavior. The investigation of possible decrease in sexual satiety mechanisms of treated rats and the analysis of brain plasticity of the altered sexual behavior remain to be elucidated in this model.

The present toxicological animal model study is expected to be relevant to human health by providing helpful information for EDC risk assessments. Even though human exposure doses for EDC are low, an average of lifetime and/or accumulative exposure can represent a significant vulnerability to EDC concentrations and their consequential effects. This is of great concern, especially in developing countries, where rigorous regulation of chlorophenols and other EDC use is immensely lacking.

## Figures and Tables

**Table 1:** The table shows plasma testosterone and estradiol average concentrations from male and female control and 2,4-DCP-treated animals.

Treatment	TESTOSTERONE ng/dL	Treatment	ESTRADIOL pg/mL
DCP	4.691	DCP	30.683
None	4.329	S/DCP	31.714

## Data Availability

The information obtained in this investigation will not be used for purposes different from research.
